# Variability in Large Language Models’ Responses to Medical Licensing and Certification Examinations. Comment on “How Does ChatGPT Perform on the United States Medical Licensing Examination? The Implications of Large Language Models for Medical Education and Knowledge Assessment”

**DOI:** 10.2196/48305

**Published:** 2023-07-13

**Authors:** Richard H Epstein, Franklin Dexter

**Affiliations:** 1 Department of Anesthesiology, Perioperative Medicine and Pain Management University of Miami Miller School of Medicine Miami, FL United States; 2 Division of Management Consulting Department of Anesthesia University of Iowa Iowa City, IA United States

**Keywords:** natural language processing, NLP, MedQA, generative pre-trained transformer, GPT, medical education, chatbot, artificial intelligence, AI, education technology, ChatGPT, Google Bard, conversational agent, machine learning, large language models, knowledge assessment

We read with interest the recent study by Gilson and colleagues [1], “How Does ChatGPT Perform on the United States Medical Licensing Examination? The Implications of Large Language Models for Medical Education and Knowledge Assessment.” Based on their detailed evaluation of the model’s performance, including content analysis and logical reasoning, the authors suggested that ChatGPT has potential application as a medical education tool to support interactive peer group education. We take no issue with those conclusions. However, what is not emphasized in the article is that search engines often provide different results based on the login credentials of the person executing the search, the location (country), and the device [2,3]. Thus, because the performance results presented by the authors did not account for this variability, their single comparisons between the various models against the different sets of questions may be statistically unreliable. Again, we are not suggesting that the authors’ useful conclusions would change, but quantitative performance will differ.

We evaluated this issue of varying responses using all questions from the most recent quarterly, online, open-book American Board of Preventive Medicine (ABPM) pilot evaluation of a longitudinal assessment program for the maintenance of certification of its clinical informatics diplomates. We evaluated ChatGPT, version 3.5 (OpenAI), and Google Bard (Alphabet Inc) by copying and pasting each of the 12 questions and the corresponding 4-part multiple-choice options into the chatbots’ message boxes on March 30, 2023, and April 1, 2023, respectively. We added a request to provide citations for each question. Both chatbots supplied the option they considered best, with a justification, references, and an explanation as to why each option was either incorrect or inferior to the recommended answer.

For ChatGPT, the series of 12 questions was performed 10 times in separate chat sessions to avoid memory effects from a previous search, with each session scored against the answer key provided by the ABPM. The results showed that out of the 12 questions, there were 9 sessions where 8 correct responses were achieved and 1 session where 9 correct responses were achieved. Although 8 questions had perfect (10/10) concordance with the answer key, there were 2 questions with 2 different answers and one with 3 different answers. There was a twelfth question where the same answer was provided for each session that disagreed with the answer key. These scores were at least as good as the average performance of the diplomates participating in the maintenance of certification process (61%, to date), which allows the use of online resources, and likely would have represented a passing score. We also evaluated the experimental ChatGPT, version 4.0, in 5 separate chat sessions, which produced sequential scores of 10, 8, 8, 6, and 7. For Google Bard, the process was performed 9 times, and the most common answer was selected as the best response. The modal responses were correct for 7 out of 12 questions (sequential scores of 7, 6, 7, 6, 7, 5, 6, 7, and 8). There were 5 questions for which 2 different answers were provided and 1 question for which all 4 answers were provided as correct answers during different sessions. Google Bard agreed with the ABPM answer key for only 4 questions in all sessions.

The questions where the large language models consistently disagreed with the ABPM answer key were either based on low-level evidence or involved an opinion on a “best” approach. As implied by Gilson et al [1], these dichotomies emphasize the importance of using artificial intelligence products to foster discussion rather than considering them an arbiter of truth. Since both ChatGPT and Google Bard provide justifications and references, groups or individuals using these products for education can learn from the supplied material. If used for such purposes, we recommend submitting questions several times in separate sessions and considering the range of responses.

## Abbreviations

ABPM: American Board of Preventive Medicine

## References

[1] Gilson A, Safranek CW, Huang T, Socrates V, Chi L, Taylor RA, Chartash D (2023). How does ChatGPT perform on the United States Medical Licensing Examination? The implications of large language models for medical education and knowledge assessment. JMIR Med Educ.

[2] Why your Google Search results might differ from other people. Google Search Help.

[3] McEvoy M (2020). Reasons Google Search results vary dramatically (updated and expanded). Web Presence Solutions.

